# Comparison of 3D printed nose bolus to traditional wax bolus for cost‐effectiveness, volumetric accuracy and dosimetric effect

**DOI:** 10.1002/jmrs.378

**Published:** 2020-02-03

**Authors:** Christine Albantow, Catriona Hargrave, Amy Brown, Christopher Halsall

**Affiliations:** ^1^ Radiation Therapy Townsville Cancer Centre Townsville Hospital and Health Service Townsville Queensland Australia; ^2^ Radiation Oncology Princess Alexandra Hospital – Raymond Tce Campus South Brisbane Queensland Australia; ^3^ Faculty of Health School of Clinical Sciences Queensland University of Technology Brisbane Queensland Australia; ^4^ Elekta Pty Ltd North Sydney New South Wales Australia

**Keywords:** 3D Printing, bolus, nose, radiotherapy

## Abstract

**Introduction:**

Three‐dimensional printing technology has the potential to streamline custom bolus production in radiotherapy. This study evaluates the volumetric, dosimetric and cost differences between traditional wax and 3D printed versions of nose bolus.

**Method:**

Nose plaster impressions from 24 volunteers were CT scanned and planned. Planned virtual bolus was manufactured in wax and created in 3D print (100% and 18% shell infill density) for comparison. To compare volume variations and dosimetry, each constructed bolus was CT scanned and a plan replicating the reference plan fields generated. Bolus manufacture time and material costs were analysed.

**Results:**

Mean volume differences between the virtual bolus (VB) and wax, and the VB and 18% and 100% 3D shells were −3.05 ± 11.06 cm^3^, −1.03 ± 8.09 cm^3^ and 1.31 ± 2.63 cm^3^, respectively. While there was no significant difference for the point and mean doses between the 100% 3D shell filled with water and the VB plans (*P*> 0.05), the intraclass coefficients for these dose metrics for the 100% 3D shell filled with wax compared to VB doses (0.69–0.96) were higher than those for the 18% and 100% 3D shell filled with water and the wax (0.48–0.88). Average costs for staff time and materials were higher for the wax ($138.54 and $20.49, respectively) compared with the 3D shell prints ($10.58 and $13.87, respectively).

**Conclusion:**

Three‐dimensional printed bolus replicated the VB geometry with less cost for manufacture than wax bolus. When shells are printed with 100% infill density, 3D bolus dosimetrically replicates the reference plan.

## Graphical abstract

This study evaluated the volumetric, dosimetric and cost differences between wax and 3D printed versions of customised bolus used in the radiotherapy treatment of the nose. Three‐dimensional printed bolus replicated the virtual bolus geometry with less cost for manufacture than wax bolus. When printed with 100% internal wall fill, 3D bolus dosimetrically replicates the reference plan.

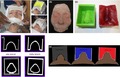

## Introduction

Research and development into three‐dimensional (3D) printer applications have grown exponentially since its development in the early 1980s.^1^ Today, 3D printer laboratories exist in many hospitals around the world, providing service to all medical streams.^2^

Three‐dimensional printing allows the conversion of digital models from data sets created via 3D ultrasound, computed tomography (CT) and magnetic resonance imaging into physical objects.^2^, ^3^ In radiotherapy, 3D printed bolus is gaining momentum as the ideal patient bolus due to its durability, and superior patient surface contact over manually manufactured wax or gel bolus.^4^, ^5^ The treatment of skin cancer requires the use of bolus to overcome the skin‐sparing effect, with the nose being one of the most difficult areas of skin to treat due to its contour changes.^9^ Nose bolus needs to cover and conform to the patient’s nose, and this ensures full dose is delivered to the skin surface as well as a homogenous dose distribution to the nose.

The process of nose bolus manufactured from wax is labour intensive. Volumetric and dosimetric accuracy is limited by the experience and skill of the radiation therapist (RT) or mould room technician, as well as variations inherent to the manual nature of the process, including air cavity flaws and imperfect contact with the patient’s skin.^6^, ^7^ Three‐dimensional printed bolus offers a unique solution to eliminate any physical variation between the bolus planned within a treatment planning system (TPS) and what would otherwise be constructed by a skilled hand, thereby limiting the potential for a difference between the planned and delivered radiation dose.^6^, ^8^

This study aimed to examine the volume, dimensional and dose differences between manually manufactured wax bolus and a 3D printed shell to the virtual bolus demonstrated in a radiotherapy plan for photon treatment to the nose, and to explore which type of bolus is more cost‐effective to produce.

## Method

### Nose bolus construction

**Figure 1:**
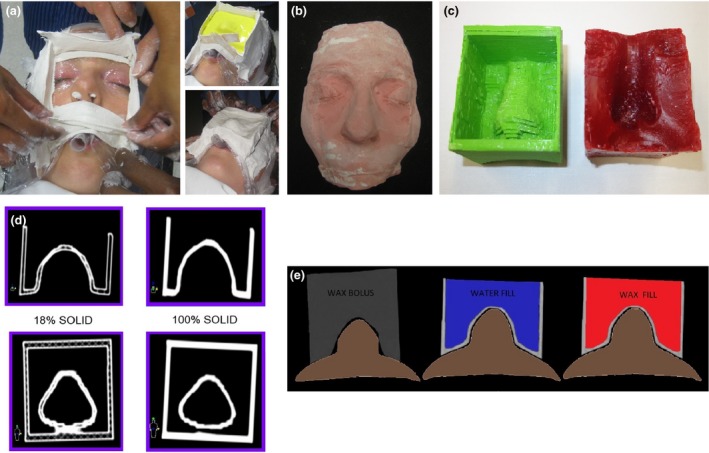
Bolus Construction: (a) Creating a plaster impression, (b) plaster impression, (c) 3D printed nose bolus without fill (green) and wax bolus (red), (d) 3D shell with varied internal fill, 18% print fill and 100% print fill and (e) Nose bolus: wax (grey), 3D print and water fill (blue), 3D print and wax fill (red).

Institutional ethics approval was granted, with informed consent given by participants. Twenty‐four volunteers from the staff group were recruited for a plaster impression of their nose and face. Alginate and Plaster of Paris were used to make the impressions (Fig. 1A) as per department protocol. Wet plaster was then poured into the negative impressions to make a positive plaster impression (Fig. 1B). Each positive plaster impression was CT scanned at 2 mm slices, imported into the Monaco TPS (Elekta, MO), contoured, and 3D planned with virtual bolus and parallel opposed 6 MV lateral fields with collapsed cone algorithm. Virtual bolus (VB) depth from the lateral edge of the nose was at least 2 cm, and a relative electron density (RED) of 1.0 assigned. The VB created for each plan was then manually manufactured in wax with the VB dimensions directly outlined on the visually levelled plaster impression, and four straight walls constructed with cardboard and plaster in which to pour the wax. The VB was then produced in 3D print (Fig. 1C). Conversion of the VB Digital Imaging and Communications in Medicine (DICOM) structure to stereolithography file format (.stl) was achieved by exporting the DICOM images and structure file to 3DSlicer with the radiotherapy extension.^9^ The converted .stl file was then uploaded into the Cura software (Ultimaker B.V., Geldermalsen, The Netherlands) for print orientation, and the 3D print completed on an Ultimaker2 + printer (Ultimaker B.V., Geldermalsen, The Netherlands). The 3D print files did not undergo any external software smoothing. Printing parameters included a layer height of 0.2 mm, external perimeter thickness of 0.5 mm and top/bottom thickness of 0.8 mm, as per the default fast draft setting in Cura. Polylactic acid (PLA) was chosen as the printing material due to its reduced particle emission and ease of use.^10^, ^11^, ^12^ A duplicate was made for each 3D printed shell to check for printing reproducibility and conformity of fit, with the internal fill density varied from 100% to 18% to reduce print time (Fig. 1D). The wall width of the 3D printed shells was a minimum of 3 mm. Print time was verified using a time‐lapse camera (set with 30‐second intervals).

### Volumetric evaluation

Potential volume variations of the manually constructed wax bolus and 3D printed bolus from the reference plan’s VB contours were assessed by CT scanning each individual constructed bolus, importing the CT and fusing the data set with the original CT scan of the volunteer impressions. All scanned wax bolus and 3D shells were contoured (external walls for the 3D shells). Two RTs completed bolus contours, one RT for the 18% 3D shells and one for the 100% 3D shells and wax bolus. The volume of each constructed nose bolus was recorded in cubic centimetres (cm^3^) and descriptive statistics calculated. Additionally, micrometre measurements were taken along the width and length of the 3D shells for comparison with the virtual equivalent. As the Shapiro–Wilk test for normality indicated all volume and micrometre data were normally distributed, the paired *t*‐test was performed to compare the manually constructed and 3D shell bolus with the reference treatment plan VB contours used to generate them. Intraclass correlation coefficients were calculated to determine the reproducibility between the volume of the constructed nose bolus and the reference VB.

### Dosimetric evaluation

The plan for each plaster impression was copied to the CT image data set for both the manually manufactured wax and the 3D shells. Dose at four defined points was recorded (A, B, C and Reference) for comparison (Table 1). As points cannot be copied across CT scans within Monaco, a sphere was created around the point, copied across the study set and a point placed within the sphere. Points A, B and C were placed as close as possible to the same position across plans, accepting differences in exact point position (real slice or between slices) and that the bolus was not perfectly straight on each scan. Additionally, a pseudo‐PTV was created on the VB plan, copied to all other bolus data sets, and maximum and mean doses to this volume recorded.

**Table 1:** Defined points at which dose was measured.

Point	Description
A	CT zero on midline (ML), defined as the reference point in 3D space at which a CT scan is acquired
B	1 cm superior to CT zero on ML
C	1 cm inferior to CT zero on ML
Reference	0.5 cm Ant to the posterior edge of the beam on CT zero ML

For additional dosimetric analysis, dose calculations were computed once on the twenty‐four 18% shells with water fill and twice on the 100% shells, once with water fill and once with wax fill (Fig. 1E). The 100% shell group with wax fill was CT scanned with wax in place, and the 18% and 100% shell group with water fill were CT scanned and a forced RED of 1.0 applied for the water fill (Fig. 1E). No other density overrides were applied to the manufactured wax or 3D printed bolus. The difference in the dose distribution for each defined point (A, B, C and reference) was calculated as well as the maximum and mean doses to the pseudo‐PTV on the constructed bolus and the original reference plan. As the Shapiro–Wilk test for normality indicated that not all of the dose difference data were normally distributed, the Wilcoxon signed‐rank test was performed to compare the dosimetry between the manually constructed and 3D printed shell bolus, with the original reference treatment plan with the VB contours. Intraclass correlation coefficients were calculated to determine the reproducibility between the dosimetry of the plans.

### Cost‐effectiveness

Cost‐effectiveness in this study focuses on consumable costs. The purchase cost of the 3D printer, maintenance and forecast replacement of durable parts were not included in the calculation; likewise, the water bath and tools for the manufacture of the wax bolus were also omitted.

Of the 48 3D prints, three prints failed to complete infull. The time lost on these initial print fails was excluded in the cost analysis.

The manufacture time and material costs for each manually constructed wax bolus were recorded. Staff time for wax bolus manufacture was calculated based on one RT with 7 years experience (RT7) and one graduate RT (RT1) working together, at RT7 = $45.36 and RT1 = $33.81 per hour. This is reflective of practice in the department where a graduate RT is mentored by a senior RT. Active staff time for 3D print construction was calculated from the time of DICOM export up to the time ‘print’ was selected on the 3D printer. Material costs of all 3D prints included the weight of PLA and single‐use dental wax in grams. The cost of water was negligible. Staff time for 3D bolus manufacture was based on the rate of one RT7 working alone.

## Results

### Volumetric evaluation

There was no statistical difference between the volumes of the VB and wax block (*P* = 0.2) and the VB and the 18% shell (*P* = 0.54), however, there was a significant difference between the VB and the 100% shell (*P* = 0.02). The intraclass correlation coefficient (ICC) values comparing VB and wax bolus, VB and 18% shell and VB and 100% shell were 0.99, 0.92 and 0.99, respectively. While there was a significant difference between the micrometre measurements of the length and width, the ICC values were 0.997, 0.996, 0.99 and 0.997 for the 18% and 100% shell widths and the 18% and 100% shell lengths, respectively, when compared to the VB.

**Figure 2:**
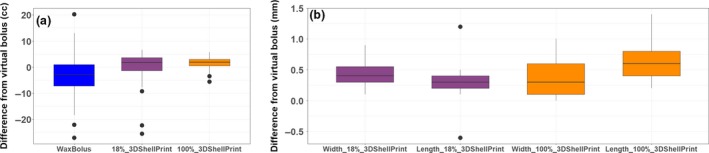
Data analysis. (a) Volume difference of wax, 18% shell 3D print and 100% shell 3D print to the virtual bolus, (b) dimensional difference of wax, 18% shell 3D print and 100% shell 3D print to the virtual bolus.

**Figure 3:**
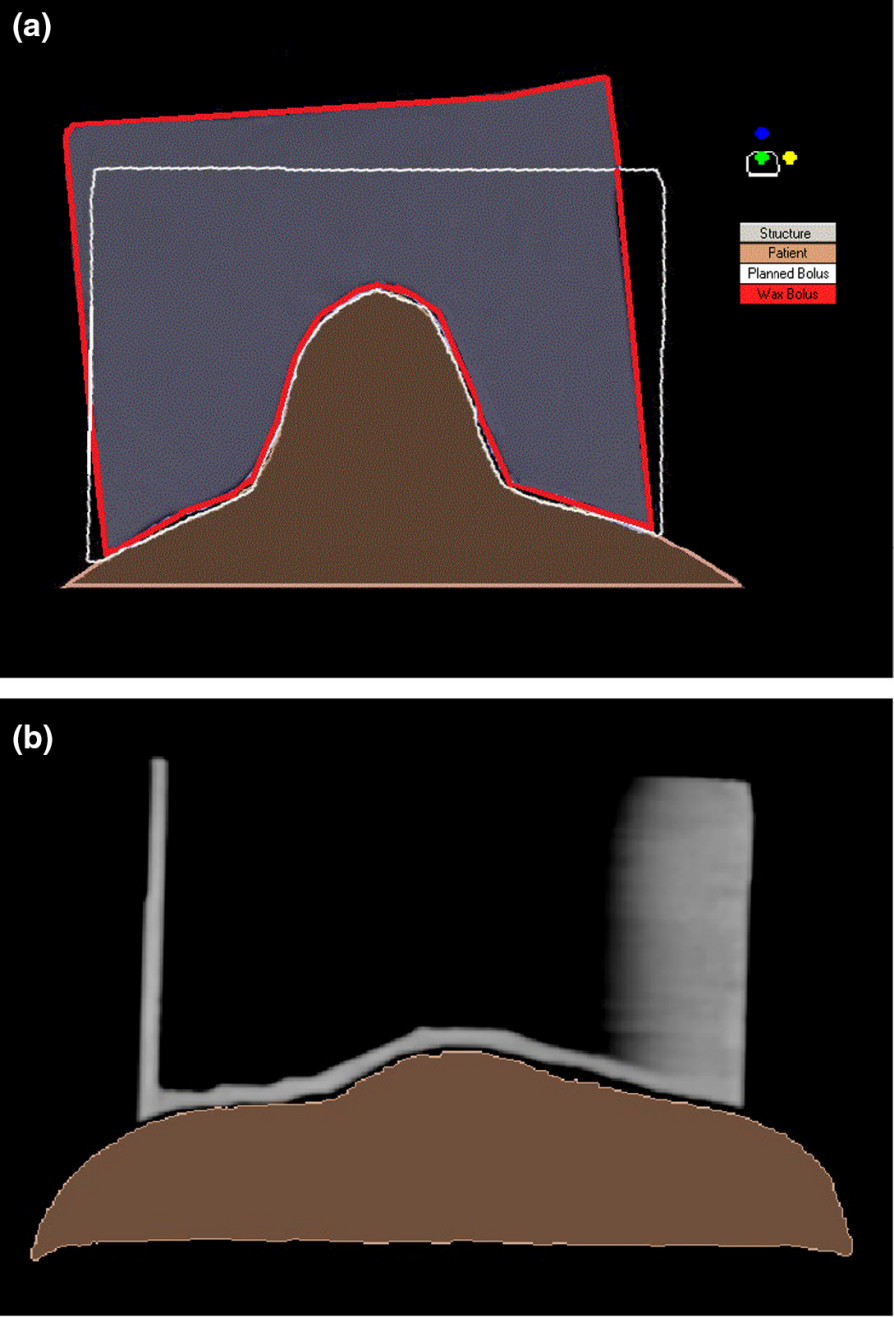
Nose bolus. (a) Difference between planned (white outline) and manufactured wax nose bolus (red outline) and (b) demonstration of an indistinct bolus edge.

The 3D printed bolus produced smaller variations from the reference plan VB compared to manually manufactured wax bolus. The mean volume difference between the reference plan VB and 18% shell was –1.03 ± 8.09 cm^3^, and 1.31 ± 2.63 cm^3^ for the 100% shell (Fig. 2A). For the physical wax bolus, the mean volume difference was −3.05 ± 11.06 cm^3^. The mean width difference between the reference plan VB and the 18% shell and 100% shell was 0.04 ± 0.02 and 0.04 ± 0.03 cm^3^, respectively, while the mean 18% shell and 100% shell length difference was 0.03 ± 0.03 and 0.03 ± 0.03 cm^3^, respectively (Fig. 2B). Across the wax bolus group, differences in tilt relative to the plaster face accounted for a mean of 71.48 cm^3^ in extra wax and 11.41 cm^3^ in missing wax across the length of the bolus (Fig. 3A). The average amount of superfluous anterior wax was 60.43 cm^3^. Only one of the 24 wax blocks did not extend anteriorly enough to match the VB but would still be considered clinically acceptable for treatment.

Volume differences were observed in the reference plan VB contour as it was duplicated from the plaster impression and applied to the CT data sets of the manufactured pieces of bolus. The average volume of the VB wall contour was 69.41 cm^3^, however, the average duplicate contour volume was 72.46 cm^3^ for the 18% shell, and 72.69 cm^3^ for the 100% shell.

### Dosimetric evaluation

**Figure 4:**
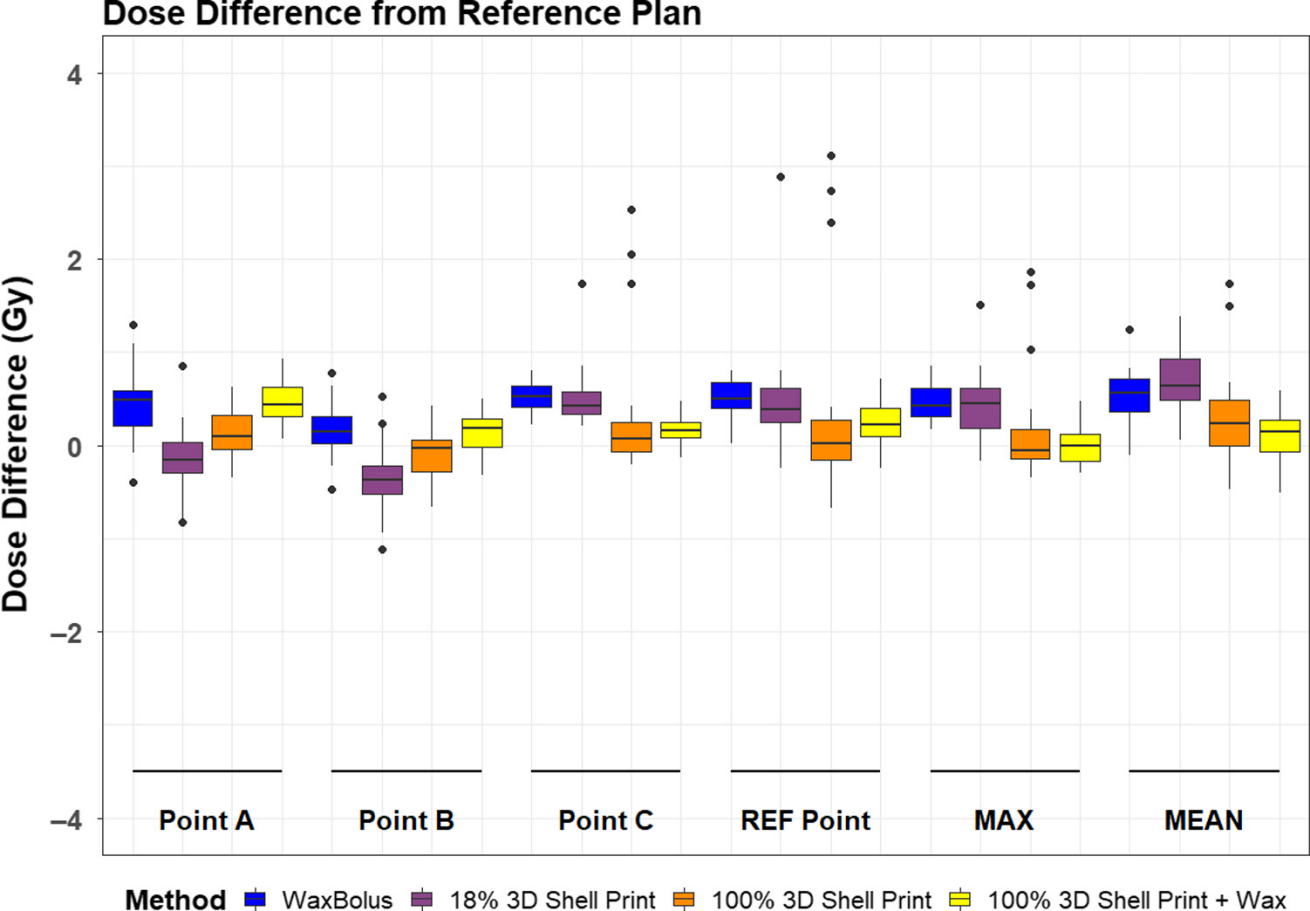
Dose point difference from reference plan. The outliers can be attributed to the same scans. The 18% shell outliers all belong to the same single subject’s plan as generated on the CT scan of their physical 18% shell nose bolus, and the outliers on the 100% shells are all from the same three volunteers plan generated on the CT scan of their physical 100% shell nose bolus.

The average RED of the PLA filament for this study was 1.08, and the average RED for the dental wax was 0.88. Boxplots of the difference between dose points, maximum and mean doses for the VB on the reference plan and the wax and 3D shells are shown in Fig. 4. There was no significant difference (*P *> 0.05) for any of the dose points and mean dose to the pseudo‐PTV when the VB and 100% shell with water fill plans were compared, as well as for the maximum point dose in the pseudo‐PTV for the 18% shell. There was also no significant difference between the 100% shell with wax fill and the VB for dose Point C (*P *> 0.05). All remaining points across each of the plans differed significantly (*P* < 0.05) from the reference plan. The ICC values comparing the virtual bolus reference plan doses with corresponding doses on the plans generated on each CT scan of the wax, 18% shell, 100% shell with water and 100% shell with wax, ranged from 0.59 to 0.79, 0.58 to 0.76, 0.48 to 0.88 and 0.69 to 0.96, respectively.

### Cost‐effectiveness

Three‐dimensional printed bolus was more cost‐effective to produce than the manually manufactured wax bolus (Table 2). See Appendices 1, 2, 3 for a specific breakdown of material and time costings and individual volunteer measures. The mean time taken to 3D print the 18% and 100% shells was 529 and 747 minutes. As the 18% shells were converted and completed prior to all the 100% shells, the total cost of staff time is higher for the 18% shell group due to staff being more familiar with the process of DICOM to .stl file conversion for the 100% shell group.

**Table 2:** Mean costs for staff time and materials for each bolus type produced.

Mean costs	Wax bolus	18% shell + water	100% shell + water	100% shell + wax
Staff time	$138.54	$10.58	$9.83	Not recorded
Materials	$20.49	$5.21	$8.27	$13.87
Total	$158.68	$16.61	$17.98	$23.84†

^†^ Total mean costs for 100% shell + wax calculated with 100% shell + water staff time, recognising that this may be underestimated.

## Discussion

### Volumetric evaluation

Precision in the manufacture of the wax bolus was heavily reliant on staff skill. To produce bolus with straight edges that will fall parallel to the incident treatment beam, the tilt of the plaster impression must precisely replicate that of the patients’ head at simulation. If the plaster impression is misaligned, then the defining borders of the wax bolus marked on the plaster impression will result in a different vertical edge than planned (Fig. 3A). This was evident in the volume variation results of the wax bolus from the reference ideal. Three‐dimensional prints overcome this issue of misalignment as they are defined as a single structure that can be printed in any orientation to achieve the same consistent result. By eliminating tilt variation with a 3D print, a more accurate final product results. Fujimoto et al.^13^ found 3D printed bolus agreed with VB within 0.02 cm.

The results showed a volume difference between the reference volume of the replicated 3D prints, and this is due to the bolus failing to be CT scanned perfectly straight so definition of the definitive borders of the bolus is open to interpretation resulting in the reference volume changing between each data set (Fig. 3B).

Visual assessment of the fusion between plaster impression and the 3D shells showed excellent results with less than a 1 mm gap across the plans (Fig. 1E). This visual assessment reflects that which would be undertaken clinically on patient verification imaging, ensuring no significant gaps. As seen in Fig. 1C, the 3D prints have a slight step between layers of the print along the nasal dorsum created by the difference between contours from one CT slice to the next. This creates a small space between skin and bolus that can be overcome in the clinical setting with a small amount of KY jelly applied to the patient’s nose. Other options involve the use of smoothing software applied to the 3D contour to remove the steps; however, care must be taken that use of such software does not change the overall size of the 3D contour in the area of smoothing. At the time of creating our 3D prints, smoothing options investigated resulted in an overall reduction in size to the area being smoothed; for this reason, no smoothing was applied.

### Dosimetric effect

There are some limitations with regard to the impact of the methods used to create the plans and dose point accuracy. Dose assessment was completed by comparing points in the same locations on the reference plans and the wax or 3D shell plans. As points cannot be copied between data sets within Monaco, there is slight variation in their placement between plans, specifically where the planned bolus has not be scanned perfectly vertical and where reproduced points are falling on or between real CT slices. The 100% shells with water came closest to matching the reference RED of the VB, with in‐house studies post‐completion of this project demonstrating a PLA print with 93.5% solid construction equivalent to a RED of 1.0.^14^ Dosimetric analyses in the literature agree with our findings.^15^ A comparison of commercial superflab to 3D printed bolus found no significant difference (<1%) when measured with gafchromic film.^5^

The 18% value for the less dense 3D prints was chosen to maintain print rigidly with a reduced overall print time. Although dose analysis was carried out on this shell group, in clinical practice this would be unacceptable as the air cavity within the print itself is counterintuitive to reducing air cavities between the nose bolus and the nose surface through improved surface contact. Assuming the largest contributing factor between the 18% and 100% shells with water fill is the density of the print itself, the increase of dose in the 18% group can be attributed to the loss of attenuation within the walls of the print. The increase in dose for the 100% print with wax fill can be attributed to the wax RED of 0.88 resulting in reduced attenuation of the beam. Ricotti et al.^16^ reported discrepancies between calculated and measured dose using gafchromic film were within 5% for both 40% and 60% infill.

### Cost‐effectiveness

The finding of our cost analysis is in line with those of Arenas et al who demonstrated reduced labour and material costs for 3D printed bolus compared to traditional bolus.^17^ Similarly, a reduction in RT staff time of 4 hours was reported by Canter et al.^15^ As the printer does not require active monitoring, staff can set the printer to run overnight or complete other tasks during the print time, thereby improving staff cost‐effectiveness. Where experienced traditional mould room skills are lacking in a department, 3D printing provides for excellence in a consistent product that can be achieved through very little real‐time training. For departments with limited staff and resources, this also opens up the possibility of outsourcing 3D printed bolus from the growing number of 3D printer companies specialising in radiotherapy products.

### Further evaluation

In conjunction with this project, PLA phantoms were produced to assess material uniformity, with geometric, dosimetric, tissue equivalence and radiation damage evaluation undertaken.^14^

### Considerations and implications

In practical terms, caution is required if employing water‐filled bolus on the linear accelerator to ensure no accidental spillage. This can be mitigated by increasing the print wall height and indicating the minimal water level required for the plan within the print.

The incidence of radiotherapy treatment to the nose at this centre is low (5–9 patients per annum) in the last 5 years. Three‐dimensional printed bolus is a viable replacement for manually manufactured wax bolus. By removing the need for a plaster impression, we significantly reduce the time a patient is required within the department and negate the need for a potentially confronting experience in plaster impression acquisition (see Appendix 4 our departmental workflows of traditional wax bolus versus 3D print bolus). In the case where a diagnostic CT is available for the patient prior to simulation, a 3D print could be generated from this CT and fit of the bolus confirmed with the planning CT rather than on the first day of treatment with kV or CBCT imaging. Optical surface scanning also provides an option for avoiding plaster bust acquisition for patients with significant claustrophobia; however, its use would not negate the need for a planning CT of the patient in a mask.^17^, ^18^

The choice to 3D print the bolus as a shell with fill rather than as a whole piece was to reduce the time of printing and the exposure of staff to released particles in the air from the process of fused deposition modelling. There are no long‐term health effect studies associated with 3D printer use in confined space.^16^

## Conclusion

Three‐dimensional printed bolus shells for photon nose treatments are more accurate than wax bolus in reproducing a virtual bolus volume from the treatment planning system. When printed in 100% shell infill density, and filled with water, 3D bolus shells accurately replicate the reference plan dosimetry. Three‐dimensional printed bolus shells produce superior bolus geometry, are more cost‐effective to produce than traditional wax bolus and do not require the same technical skills for production as traditional wax bolus. This research supports the move to 3D printed bolus for the nose and will significantly reduce patient appointment times, leading to an improved patient experience. Further investigations are necessary for 3D print bolus for different modalities such as electrons and for different body sites.

## Conflict of interest

The authors declare no conflict of interest.

## Appendix 1

### Material and time costings

**Table:**

Project materials and costings	
Product	Cost (AUD)
Alginate	$0.04 per g
Wax	$0.76 per sheet
Printer filament: PLA	$0.09 per g
Plaster of Paris	$0.65 per m
Tongue depressor	4 cents per depressor
Soft paraffin wax	$0.01 per g
Cling film	$0.13 per m
Micropore	$0.57 per roll
Box of tissues	$0.01 per tissue
Water	$0.003 per L
Linen	$1.51 per kg
Hand moisturiser	$0.01 per g
Plaster	$0.01 per g
Vaseline petrolatum gauze strip	$3.58 per packet
Sticky tape	$0.04 per m

**Table:**

Manufacturing bolus costs	
Process	Mean cost (AUD)
Wax	
Acquiring plaster nose impression	$5.62
Making the plaster face	$3.28
Making wax bolus	$11.41
18% shell 3D print	
PLA filament	$5.21
100% shell 3D print	
PLA filament	$8.27
100% shell 3D print with wax fill	
PLA filament	$8.27
Wax	$5.64
Water	Negligible

**Table:**

Project time record	
Process	Time (minutes)
Wax	
Time taken to acquire nose impression	27
Time taken to make the plaster face	17
Time taken to make wax bolus	59
18% shell 3D print	
Active therapist time	14
100% shell 3D print	
Active therapist time	13
